# The Therapeutic Potential of Polyphenols in Modulating Barrier Lipids, Microbiome Interactions, and Inflammatory Pathways in Atopic Dermatitis

**DOI:** 10.3390/nu18091365

**Published:** 2026-04-25

**Authors:** Karolina Blady, Bartosz Pomianowski, Leon Smółka, Miłosz Strugała, Karolina Kursa, Agata Stanek

**Affiliations:** 1 Student Scientific Association, Department of Internal Medicine, Metabolic Diseases, and Angiology, Faculty of Health Sciences, Medical University of Silesia, Ziolowa 45/47 St., 40-635 Katowice, Poland; s83086@365.sum.edu.pl (B.P.); s81350@365.sum.edu.pl (L.S.); s83152@365.sum.edu.pl (M.S.); s91506@365.sum.edu.pl (K.K.); 2 Department of Internal Medicine, Metabolic Diseases, and Angiology, Faculty of Health Sciences in Katowice, Medical University of Silesia, Ziolowa 45/47 St., 40-635 Katowice, Poland; 3 Upper-Silesian Medical Center, 45/47 Ziołowa St. 40-635 Katowice, Poland

**Keywords:** polyphenols and dermatological benefits, atopic dermatitis, skin diseases, inflammatory skin conditions and diet, antioxidants and skin function

## Abstract

Atopic dermatitis (AD) is a chronic inflammatory skin disease with a complex pathogenesis involving epidermal barrier dysfunction, microbiome dysbiosis, and immune dysregulation. Despite significant advances in therapy, including biologics and targeted treatments, their use may be limited by adverse effects, highlighting the need for safe adjunctive strategies. Polyphenols are naturally occurring bioactive compounds that are abundant in plant-based foods and are known for their anti-inflammatory, antioxidant, and immunomodulatory properties, making them promising candidates for supportive AD management. This review integrates current evidence on the effects of polyphenols on epidermal barrier lipids, microbiome interactions, and key inflammatory pathways, including NF-κB and JAK/STAT signaling. Additionally, the role of polyphenols in modulating dendritic cell and neutrophil activity, and reducing reactive oxygen species (ROS) production and neutrophil extracellular trap (NET) formation, as well as their potential involvement in mitophagy regulation, is discussed. Polyphenols support epidermal barrier integrity by modulating the expression of key structural proteins, including filaggrin, involucrin, and loricrin, leading to a reduction in transepidermal water loss (TEWL). Furthermore, they interact bidirectionally with the gut microbiome, acting as metabolic substrates for beneficial bacteria and promoting the growth of short-chain fatty acid (SCFA)-producing species such as *Lactobacillus*, *Bifidobacterium*, and *Akkermansia*, while simultaneously inhibiting pathogenic strains. These findings highlight the role of polyphenols in maintaining microbiome homeostasis and supporting epidermal barrier integrity. The review encompasses findings from clinical studies, animal models, and mechanistic investigations, while also addressing limitations related to polyphenol bioavailability. Overall, polyphenols may represent a valuable adjunctive approach in AD management; however, further well-designed clinical and mechanistic studies are required to confirm their therapeutic potential.

## 1. Introduction

Atopic dermatitis (AD) is a chronic condition characterized by periods of exacerbation and remission [1,2]. Lesions typically localize on the face, neck, in the elbow and knee creases, and on the dorsal surface of the hands in adults [3]. Symptoms include pruritus, erythema, lichenification, and scaly plaques of skin. In individuals with AD, the skin has a weakened barrier due to lipid abnormalities and transepidermal water loss [4,5,6]. A key feature of these disturbances is the alteration in the lipid composition of the stratum corneum (ceramides, cholesterol, and free fatty acids (FFAs)), which exacerbates transepidermal water loss (TEWL) and promotes allergen penetration and microbial colonization [7,8]. It may seem that epidermal barrier disturbances affect only the skin involved in the disease process, but such disturbances can also involve skin that appears free of inflammation [9]. A characteristic feature of AD is damage to the stratum corneum, allowing pathogen invasion and increasing susceptibility to irritants [10]. This leads to skin microbiome dysbiosis, which plays a significant role in the pathogenesis of AD, affecting both the function of the epidermal barrier and the modulation of the immune response [11]. The aforementioned symptoms impact patients’ lives, and the bothersome pruritus may also occur at night, reducing sleep quality [12,13]. Patients may experience difficulties in establishing interpersonal relationships and suffer from social stigmatization [14,15]. It has been observed that depression and anxiety symptoms occur in individuals with AD significantly more often than in healthy individuals. Therefore, the patient requires comprehensive care in this regard as well [16,17]. Treatment of AD includes topical and systemic therapies, including the use of glucocorticosteroids (GCs) and oral immunosuppressive drugs, as well as new targeted and biological drugs. Despite their effectiveness, these therapies may be associated with adverse effects and do not always provide long-term disease control, highlighting the need for safe and well-tolerated adjunctive strategies based on a better understanding of the disease’s pathogenesis [18,19,20]. The implementation of polyphenols as adjunctive therapy in skin diseases, including atopic dermatitis, requires further research; however, current evidence suggests that they may represent a valuable addition to existing treatment strategies.

**Figure 1:**
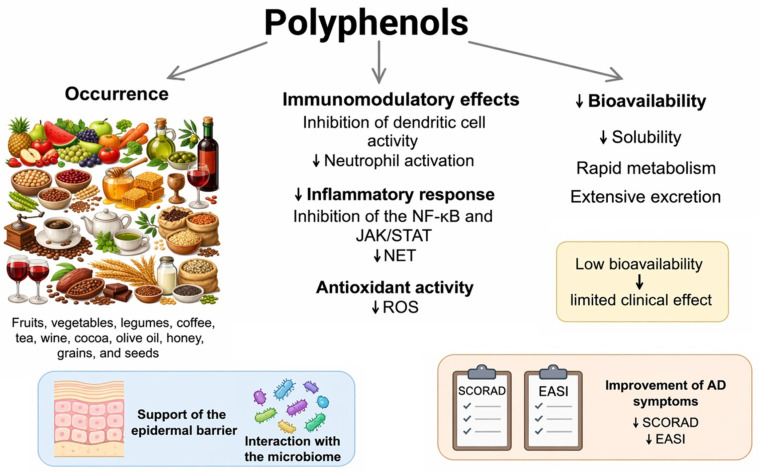
Overview of the occurrence, biological mechanisms, and limitations of polyphenol bioavailability. Polyphenols are naturally present in plant-based products and exhibit immunomodulatory, anti-inflammatory, and antioxidant properties. They can inhibit the activity of dendritic cells and neutrophils, suppressively affect the nuclear factor kappa B (NF-κB) and Janus kinase/signal transducer and activator of transcription (JAK/STAT) signaling pathways, limit the formation of extracellular neutrophil traps (NETs), and reduce the production of reactive oxygen species (ROS). However, the clinical efficacy of polyphenols may be limited due to their low bioavailability, stemming from their poor solubility, rapid metabolism, and rapid excretion. Arrows indicate the direction of changes (↓ decrease) [24,25,26,27,28,29].

Polyphenols are a group of natural bioactive compounds used in dermatology, both as oral supplements and topical preparations [21], and are found in fruits, vegetables, legumes, coffee, tea, wine, cocoa, olive oil, honey, grains, and seeds [22,23]. They have antioxidant and anti-inflammatory properties but are characterized by low solubility, rapid metabolism, and rapid excretion, resulting in low bioavailability [24,25]. The instability of polyphenols limits their therapeutic action, so a main goal of research is to increase their bioavailability, which will facilitate the translation of their biological potential into clinical practice [26,27]. Polyphenols exhibit multifaceted immunomodulatory effects, including inhibiting dendritic cell activation, limiting antigen presentation, and modulating the inflammatory response, reducing the recruitment and activation of neutrophils. Furthermore, these compounds reduce excessive production of reactive oxygen species (ROS) and inhibit the formation of extracellular neutrophil traps (NETs), which contribute to limiting tissue damage and inflammation [28]. Polyphenols inhibit the nuclear factor kappa B (NF-κB) and Janus kinase/signal transducer and activator of transcription (JAK/STAT) pathways, leading to a reduction in inflammation by limiting pro-inflammatory cytokines and inflammatory mediators [29]. Polyphenols may improve clinical outcomes, as reflected by reductions in SCORAD (Scoring Atopic Dermatitis) and EASI (Eczema Area and Severity Index) scores. Additionally, oral supplementation with polyphenols is characterized by a high safety profile and good tolerance in patients with AD [30] (Figure 1). Although polyphenols are considered safe, emerging evidence indicates that they may also exert dose-dependent adverse effects, including thyroid function interference, reduced iron absorption, and potential interactions with medications. In this review, we synthesize the current data on the impact of polyphenols on epidermal barrier lipids, interactions with the microbiome, and key inflammatory pathways in AD. The aim of this paper is a comprehensive analysis of the metabolism, mechanisms of action, and therapeutic potential of polyphenols, with particular emphasis on the results of clinical studies, animal models, and future research directions.

## 2. Materials and Methods

**Figure 2:**
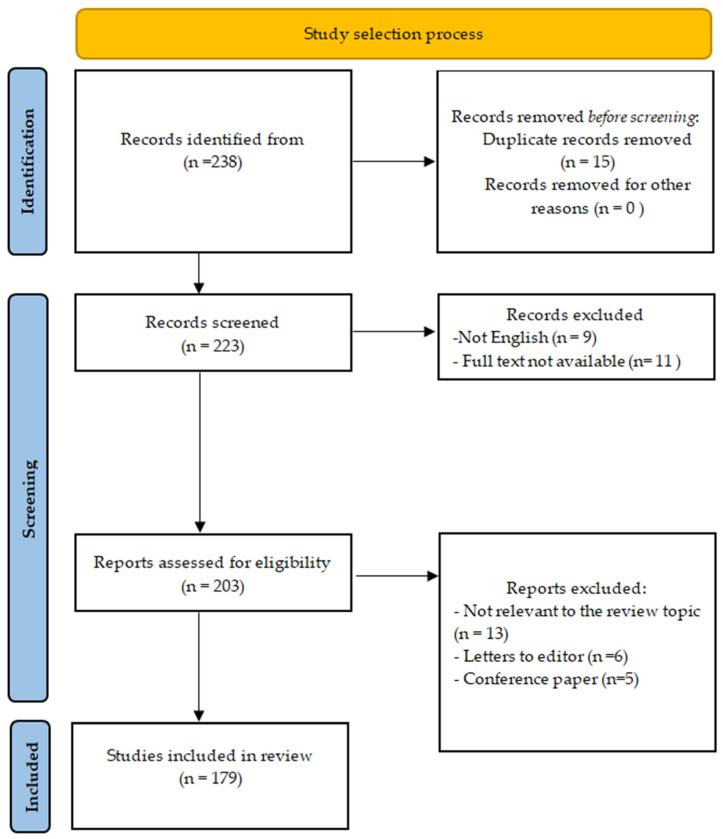
Flow chart illustrating the study selection process for publications on polyphenols in AD.

A literature review was conducted in the PubMed, Scopus, and Web of Science databases, covering publications available up to February 2026 regarding the impact of polyphenols on AD. The search was based on keywords and their combinations, including “atopic dermatitis” and “polyphenols”, in order to synthetically present the current knowledge on the immunomodulatory role of polyphenols in AD. The analysis included original articles, review papers, and meta-analyses, as well as randomized and observational clinical studies, published in English. Both studies involving patients with AD and animal and cell models providing mechanistic data were considered. Conference abstracts, publications without full text, non-English articles, and studies not directly related to atopic dermatitis or lacking relevant mechanistic or clinical data on polyphenols were excluded from the review (Figure 2). This review was conducted as a narrative synthesis to provide a comprehensive and integrative overview of the available evidence on polyphenols in AD.

## 3. Metabolism and Mechanism of Polyphenols

Polyphenols influence the modulation of AD severity through a range of mechanisms, including improving the integrity of the epidermal barrier, restoring the skin microbiological balance, and modulating inflammatory pathways [31]. This translates into a reduction in the severity of symptoms occurring in the course of AD, including pruritus and sleep disturbances [32,33]. Polyphenols commonly found in food require a series of metabolic processes essential for their bioactivation [34]. In the context of atopic dermatitis, the biological activity of polyphenols should be interpreted through three key disease-specific domains: epidermal barrier dysfunction, microbiome dysbiosis, and Th2-driven inflammation.

### 3.1. Microbiome Interactions

A proper bacterial microbiome is crucial for the metabolism of polyphenols. Polyphenols present in food require a series of metabolic processes, including esterification, acylation, glycosylation, hydrolysis, sulfation, and glucuronidation, in order to be converted into their bioactive derivatives [35]. However, a proper microbiome ensures the appropriate intestinal metabolism of polyphenols available in food, while polyphenol metabolites also modulate the composition and metabolic activity of the microbiome [36]. A study by Tao et al. [37], investigating the impact of proanthocyanidins on the gut microbiome, showed that these flavonoids promote the growth of beneficial bacteria that produce short-chain fatty acids (SCFAs), such as *Lactobacillus*, *Bifidobacterium*, and *Akkermansia*. Proanthocyanidins are metabolic substrates for these bacterial species as they possess the appropriate enzymes for their metabolism. Polyphenols, acting as prebiotics, selectively stimulate the growth of beneficial gut bacteria, leading to the inhibition of the growth of pathogenic strains, including *H. pylori*, *E. coli*, and *Salmonella* [38]. The effectiveness of polyphenols in promoting the development of beneficial bacteria that produce SCFAs has been confirmed in animal models. He et al. [39], in a study conducted on a mouse model, demonstrated the effect of polyphenols in modifying the gut microbiome by examining the *Firmicutes*/*Bacteroidetes* ratio, which is an important indicator of gut dysbiosis. The intervention involved supplementing mice with polyphenols found in apple peel, showing a significant increase in the diversity of the gut microbiome in mice, along with an increase in SCFAs and a reduction in the *Firmicutes*/*Bacteroidetes* ratio. The findings suggest a positive effect of polyphenol supplementation in maintaining a healthy gut microbiome while also indicating that it is a bidirectional intervention, emphasizing that not only does a proper microbiome influence the correct metabolism of polyphenols obtained from food, but also that polyphenols and their metabolites promote the development of beneficial gut bacteria.

**Figure 3:**
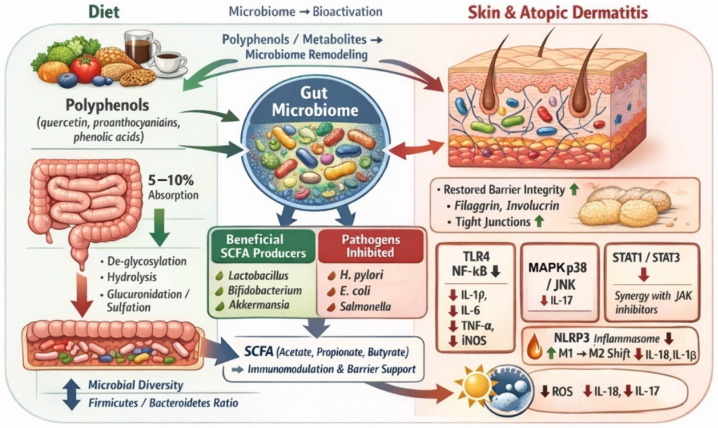
Effect of polyphenols on the gut and skin microbiome in atopic dermatitis (AD). Dietary polyphenols modulate the gut microbiota by promoting beneficial short-chain fatty acid (SCFA)-producing bacteria and inhibiting pathogenic microorganisms. Microbial metabolism of polyphenols increases SCFA production and supports gut barrier integrity and immunomodulation. Through the gut–skin axis, these changes contribute to improved epidermal barrier function, including the increased expression of filaggrin, involucrin, and tight junction proteins. Polyphenols also attenuate inflammatory signaling pathways, including TLR4/NF-κB, MAPK p38/JNK, and STAT1/STAT3, resulting in reduced oxidative stress, NLRP3 inflammasome activation, and pro-inflammatory cytokine production. Collectively, these effects may help restore microbial homeostasis and alleviate inflammation in atopic dermatitis. Abbreviations: AD—atopic dermatitis; IL—interleukin; iNOS—inducible nitric oxide synthase; JAK—Janus kinase; JNK—c-Jun N-terminal kinase; MAPK—mitogen-activated protein kinase; NF-κB—nuclear factor kappa B; NLRP3—NOD-like receptor family pyrin domain containing 3; ROS—reactive oxygen species; SCFA—short-chain fatty acid; STAT—signal transducer and activator of transcription; TLR4—Toll-like receptor 4; TNF-α—tumor necrosis factor alpha. Arrows: ↑ increase/activation, ↓ decrease/inhibition.

Polyphenols may reduce the severity of AD, including through the gut–skin axis, and their positive effects have been demonstrated for other skin diseases, including acne [40]. A proper gut microbiota plays a key role in these processes. Its correct composition enables the conversion of polyphenols available in food into active metabolites that exhibit high biological activity. In normal intestinal flora, bacteria such as *Lactococcus lactis*, *Lacticaseibacillus rhamnosus*, and *Lacticaseibacillus casei* convert polyphenols present in cereals into bioactive metabolites, such as 4-vinylguaiacol and catechol [41]. Key polyphenols include quercetin, which is commonly found in vegetables and fruits such as bell peppers, arugula, fennel, and capers [42]. Quercetin, like other polyphenols, is absorbed in both the small intestine (5–10%) and large intestine (90–95%). A series of metabolic processes occurring in the large intestine under the influence of intestinal bacteria allow the conversion of quercetin into bioactive derivatives, such as 3,4-dihydroxyphenylacetic acid [43], protocatechuic acid [44], 4-hydroxy-3-methoxybenzoic acid [45], and homoprotocatechuic acid [46]. The importance of the gut microbiota in the processes of quercetin transformation is highlighted by Kasahara et al. [47]. In a study conducted on mice, the protective effects of quercetin and its derivatives on the development of atherosclerosis were shown to be absent in mice whose intestines were sterilized. In mice with an intact gut microbiome, quercetin supplementation led to an increase in the plasma concentration of quercetin metabolites, such as benzylglutamine acid and 3,4-dihydroxybenzoic acid. At the same time, higher plasma concentrations of these metabolites negatively correlated with the severity of atherosclerotic changes in the vessels. This study underscores the importance of the gut microbiota in the transformation of quercetin into benzylglutamine acid and 3,4-dihydroxybenzoic acid. These metabolites may also modulate the intensity of inflammation in AD. It has been shown that 3,4-dihydroxybenzoic acid exhibits a range of protective actions, including exerting a photoprotective effect on the skin, preventing UVB-induced skin damage, reducing ROS levels in keratinocyte lines, and preventing UVB-induced cell apoptosis by lowering c-Fos and Jun expression [48]. A study by Yang et al. [49] investigated the therapeutic efficacy of glycosylated flavonols, namely, quercitrin, isoquercitrin, and rutin, in the treatment of AD. These glycosylated quercetin derivatives effectively reduced the expression of pro-inflammatory cytokines and chemokines, including interleukin-6 (IL-6), CXCL1 (C-X-C Chemokine Ligand 1), IL-8, TARC (thymus- and activation-related chemokine), and RANTES (Regulated on Activation, Normal T-cell Expressed and Secreted). This study highlights the therapeutic potential of these glycosylated quercetin derivatives, which undergo de-glycosylation in the intestine and are then metabolized into phenolic acids, whose anti-inflammatory effects are also a significant object of study [50]. Phenolic acids also modulate the gut microbiome, promoting the development of anti-inflammatory gut bacteria, thus reducing the severity of inflammatory bowel diseases. Another study by Han et al. [51] investigated the effects of several commonly occurring phenolic acids in food and demonstrated their impact on the severity of inflammatory bowel diseases, outlining the mechanisms of each. Chlorogenic acid, commonly found in coffee [52], exhibited an inhibitory effect on the activation of the NLPR3 inflammasome, leading to the promotion of macrophage transformation from the M1 to M2 phenotype, which resulted in reduced production of IL-1β and IL-18. This process is particularly important in autoimmune diseases, where excessive activation of M1 macrophages is associated with the exacerbation of disease symptoms [53]. Another relevant phenolic acid, ferulic acid (FA), is commonly found in cereals, chocolate, and some vegetables. Although ferulic acid has been studied in various non-dermatological conditions, its relevance to atopic dermatitis is primarily related to its ability to inhibit NF-κB and MAPK signaling pathways, which are directly involved in Th2-driven inflammation and epidermal barrier dysfunction [54,55,56]. Ferulic acid plays a significant anti-inflammatory role through the inhibition of these pathways, resulting in a reduction in the transcription of genes encoding pro-inflammatory cytokines (IL-1β, IL-6, and tumor necrosis factor alpha (TNF-α)) as well as enzymes such as iNOS (inducible nitric oxide synthase). FA also inhibits the phosphorylation of p38 MAPK (mitogen-activated protein kinase) and JNK (c-Jun-N-terminal kinase), leading to the suppression of the inflammatory response. This process is particularly important in AD, where excessive MAPK activity results in increased synthesis of IL-17, leading to the reduced expression of filaggrin and involucrin [57], which contributes to the exacerbation of AD [58]. FA also reduces the phosphorylation of STAT1 and STAT3, kinases crucial for the intensification of the inflammatory process in AD [59]. The inhibitory effect of FA on the activation of STAT1 and STAT3 could provide significant support for AD therapy using drugs that are JAK inhibitors [60]. Other polyphenols, such as baicalin, found in the root of *Scutellaria baicalensis*, also exhibit a mechanism that inhibits the phosphorylation of JAK1, STAT1, STAT2, STAT3, STAT5, and STAT6, showing the potential of polyphenols in the anti-inflammatory action in AD [61]. FA also increases the expression of antioxidant enzymes, including SOD (superoxide dismutase), glutathione peroxidase, and catalase [62,63]. An increased expression of these genes is associated with more intensive elimination of ROS, reducing the negative impact of ROS on the severity of inflammation as well as affecting the course of AD (Figure 3) [64].

An important mechanism determining the effectiveness of polyphenols in alleviating disease symptoms is their impact on modifying the epidermal barrier. By influencing the expression of proteins crucial for the proper functioning of the epidermal barrier, including filaggrin, involucrin, and loricrin, polyphenols significantly reduce transepidermal water loss (TEWL). In a study by Heinrich et al. [65], the positive effect of green tea consumption—specifically through the polyphenols that green tea contains, especially catechins—was demonstrated. After 12 weeks of an appropriate diet, it was shown that TEWL in individuals consuming green tea regularly was reduced by 12%. This highlights the importance of polyphenols in the proper functioning of the epidermal barrier.

### 3.2. Anti-Inflammatory and Immunomodulatory Mechanisms

In the pathogenesis of AD, the T helper type 2 (Th2) cell activation axis plays an important role, especially through the cytokines that activate this axis, namely, IL-4, IL-5, and IL-13 [66]. Jafarinia et al. [67] investigated the effect of quercetin and tannic acid (TA) on specific cytokines related to the Th2 response. They demonstrated the effect of these polyphenols in reducing the expression of cytokines such as IL-4, IL-5, and thymic stromal lymphopoietin (TSLP), as well as chemokines such as TARC. These results may indicate the significant role of polyphenols in modifying the Th2 activation pathway and represent an important mechanism in limiting the exacerbation of AD.

Polyphenols also influence dendritic cell (DC) activation, which plays a significant role in AD [68]. DCs are an important element promoting the Th2-directed response. Polyphenols, through the activation of Nrf2, the improvement of epidermal barrier function, and the activation of AMPK (5′AMP-activated protein kinase), reduce the activation of DCs and limit the severity of inflammation in AD [69,70].

### 3.3. Epidermal Lipid Metabolism and Barrier Function

A particularly important element in the pathogenesis of AD is alteration in the lipid composition of the stratum corneum, including ceramides, cholesterol, and free fatty acids [71]. The positive effect of polyphenols on the course of AD is also associated with their influence on ceramide synthesis pathways. In a properly functioning epidermal barrier, ceramide subclasses, including (esterified ω-hydroxy fatty acid-sphingosine) EOS, (non-hydroxy fatty acid-phytosphingosine) NP, and (α-hydroxy fatty acid-phytosphingosine) AP, are present in the correct proportions. In AD, there is a significant decrease in the proportion of long-chain ceramides, especially EOS [72], as well as a disruption in the ratio of AP to NP [73]. This leads to increased TEWL and facilitates allergen penetration [74]. The synthesis of these key lipids in keratinocytes depends on the efficiency of specific enzymatic pathways. The first rate-limiting step in the de novo sphingolipid synthesis pathway is the reaction catalyzed by serine palmitoyltransferase (SPT). Fatty acid elongases (ELOVL), which are responsible for synthesizing very-long-chain fatty acids essential for forming ceramide structures such as EOS, are also crucial. In AD, there is an increase in Th2-dependent inflammatory responses, which inhibits the expression of genes encoding ELOVL and SPT, resulting in the decreased synthesis of ceramides produced by these enzymes [75,76]. The action of polyphenols, which involves limiting the expression of pro-inflammatory cytokines, including IL-4, IL-5, and IL-13, leads to an increase in the expression of enzymes responsible for ceramide synthesis [77]. Additionally, polyphenols like resveratrol directly stimulate signaling pathways such as S1P, thereby stimulating lipid production and promoting keratinocyte differentiation [78]. The action of polyphenols and their modulatory influence on the activity of enzymes responsible for ceramide synthesis constitutes an important element in reinforcing the epidermal barrier and may, in the future, serve as a crucial therapeutic element modulating the course of AD.

Taken together, these findings indicate that polyphenols exert multidimensional effects in AD through three key pillars: modulation of inflammatory pathways, regulation of the gut microbiome, and improvement of epidermal barrier integrity.

## 4. Animal Models

Animal models play a significant role in studies investigating the impact of polyphenols on the course of AD. In murine models, AD was induced using trimellitic anhydride (TMA), 2,4-dinitrochlorobenzene (DNCB), or oxazolone, with the use of the transgenic hIL-4/hIL-4Rα (human interleukin-4/human interleukin-4 receptor alpha) double knock-in mouse model, in which the mouse IL-4 and IL-4Rα genes were replaced with their human counterparts [79,80,81]. A key observation noted in studies on animal models was a significant increase in the expression of proteins essential for the formation of the stratum corneum, especially involucrin, loricrin, and filaggrin, as well as the role of polyphenols in restoring the proper expression of these proteins at the site of skin lesions. In a study by Yang et al. [82] involving a preclinical AD model conducted in NC/Nga mice, ethanol extract from potato peel, *Solanum tuberosum* L. cv Jayoung, was administered orally to mice, in which AD-like symptoms were induced using the dust mite allergen Dfb (Dermatophagoides farinae body). The use of this polyphenol-rich extract was associated with increased filaggrin expression and a reduction in pro-inflammatory cytokine expression in mice. Proper ceramide synthesis is also necessary for maintaining the integrity of the epidermal barrier. Among polyphenols, resveratrol stimulates the sphingosine-1-phosphate (S1P) signaling pathway [83] and reduces the production of pro-inflammatory cytokines, reduces oxidative stress, neutralizes free radicals, inhibits NF-κB activation, alleviates harmful effects of UV radiation, and improves epidermal barrier function [84]. The concomitant colonization of the skin by *S. aureus* also plays an important role in the pathogenesis of AD. In studies using animal models, Nrf2−/− (nuclear factor erythroid 2-related factor 2) mice infected with *S. aureus* showed delayed expression of HO-1 (heme oxygenase) and SOD (superoxide dismutase) compared to wild-type (WT) mice. Nrf2−/− mice also exhibited increased levels of pro-inflammatory cytokines, including TNF-α, IL-1β, and CCL2. Therapeutic interventions based on polyphenol administration were associated with increased Nrf2 activation, leading to increased expression of SOD and HO-1 and reducing the severity of inflammation [85].

In animal models, the effect of pterostilbene (PTN), a resveratrol analog, was also significant. In mice with DNCB-induced AD, the use of PTN was associated with a reduction in IgE levels, pro-inflammatory cytokines (IL-4, IL-6, TNF-α), and NF-κB in the skin, leading to a reduction in symptom severity [86]. In another study using an inbred homozygous BALB/c (Bagg Albino Laboratory strain B/subline c) mouse line, oral administration of quercetin led to a decrease in histamine levels and pro-inflammatory cytokines (interleukin (IL)-6, IL-4, TSLP, interferon-γ (IFN-γ), and IL-17A) in the serum of mice with induced AD [87]. The effect of polyphenols in modifying the severity of AD was also confirmed by Huang et al. [88], who examined the effect of a topical application of oleuropein, a polyphenol found in *Olea europaea* leaves. It was found that oleuropein significantly reduced the expression of pro-inflammatory cytokines (IL-4, IL-5), promoting the development of Th2 cells, which are crucial in the pathogenesis of AD. This intervention led to a reduction in COX-2 expression, the inhibition of pro-inflammatory cytokine synthesis, a decrease in IgE levels in serum, and a reduction in mast cell and eosinophil infiltration. In the study by Tang et al. [89], a supplementation with epigallocatechin gallate (EGCG), a key polyphenol found in green tea, was administered to mice with induced atopic dermatitis. Western blot, in silico, and immunofluorescence analyses showed that EGCG, by binding to Keap1 (Kelch-like ECH-associated protein 1), disrupted its interaction with Nrf2, which resulted in the increased expression of antioxidant genes and the increased activity of catalase, glutathione peroxidase, and HO-1. Additionally, EGCG led to a reduction in the severity of inflammatory changes, decreased IgE levels, reduced ROS and MDA (malondialdehyde, a marker of oxidative stress and a product of lipid peroxidation), and reduced TEWL, which indicates improved epidermal barrier integrity. However, it should be noted that the available animal studies are highly heterogeneous in terms of experimental models (e.g., DNCB, DNFB, oxazolone), types and sources of polyphenols, routes of administration, and evaluated outcome measures. This heterogeneity limits direct comparability between studies and should be considered when interpreting the overall findings. The data presented are promising and show the significant effect of polyphenols in limiting the severity of AD in animal models; however, their direct translation to human AD remains limited. Further clinical studies conducted on patients with AD are necessary to confirm the relationships observed in animal models (Table 1). The findings are partially consistent with the limited clinical observations suggesting improvement in disease severity; however, the magnitude of effects observed in animal models is not fully replicated in human studies.

## 5. Polyphenols and Their Influence on the Clinical Features of Atopic Dermatitis

**Figure 4:**
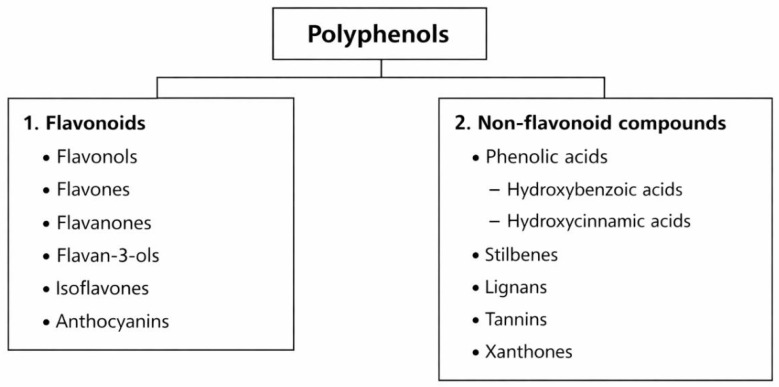
Classification of polyphenols into flavonoids and non-flavonoid compounds.

Polyphenols are active compounds found in fruits, vegetables, seeds, herbs, and algae, and their functions in plants include pigmentation, protection against harmful UV radiation and pathogens, and participation in growth [90,91,92]. They have antioxidant, anti-inflammatory, and antibacterial properties, making them potentially helpful in the treatment or prevention of diseases in humans [93]. Polyphenols are classified into flavonoids (flavonols, flavones, flavanones, flavan-3-ols, isoflavones, and anthocyanins) and non-flavonoid compounds, including phenolic acids (hydroxybenzoic acids, hydroxycinnamic acids), stilbenes, lignans, tannins, and xanthones (Figure 4) [94]. It should be noted that the majority of the evidence discussed in this section is derived from preclinical studies, and direct clinical relevance remains to be established. Although several subclasses of polyphenols have been identified, not all have been studied in the context of AD. The following sections focus on those for which mechanistic or preclinical evidence is available (Table 2).

### 5.1. Flavonoids

Flavonols are a subgroup of flavonoids, with representatives such as quercetin, kaempferol, and myricetin [95]. Quercetin is widely distributed in plant-based products, including fruits (especially apples), leafy green vegetables, seeds, buckwheat, nuts, flowers, asparagus, broccoli, onions, olive oil, green tea, and red grapes [96,97]. In an in vitro model using human keratinocytes, it was shown that quercetin reduced the expression of IL-1β/IL-6/IL-8 and TSLP, while increasing the mRNA transcription of epidermal barrier markers—occludin and E-cadherin—and simultaneously inhibiting the extracellular signal-regulated kinase 1 (ERK1) and ERK2 signaling and NF-κB [98]. Kaempferol is found in tea, cruciferous vegetables, and legumes, among other products. In a murine AD model using BALB/c mice, it was shown that kaempferol can weaken T-cell activation (by reducing the expression of cluster of differentiation 69 (CD69)). This compound also reduces ROS levels and modulates key inflammatory signaling pathways. Animal studies showed an improvement in epidermal barrier function with the administration of flavanols, characterized by decreased TEWL, reduced expression of TSLP and IL-4/IL-13, and increased expression of barrier proteins [99]. Clinical studies evaluating the effectiveness of isolated flavonols in the treatment of atopic dermatitis in humans have not been described in the cited sources. Compounds such as quercetin, kaempferol, and myricetin reduce histamine release. Additionally, their impact on microcirculation in the skin may support regenerative processes and epidermal barrier reconstruction [100]. Collectively, these data suggest that flavonols may modulate both the Th2 inflammatory response and the integrity of the epidermal barrier. Among flavonols, quercetin appears particularly relevant due to its combined effects on the inflammatory pathways and epidermal barrier integrity.

Flavones are a group of flavonoids that include apigenin and luteolin. Apigenin is present in parsley, chamomile, celery, onions, green pepper, and spinach [101,102], while luteolin is commonly found in many plants, especially in herbs such as peppermint, thyme, and rosemary [103]. In a BALB/c mouse model, it was shown that apigenin reduced the levels of cytokines associated with pruritus, such as IL-31 and IL-33, by modulating the MAPK and JAK/STAT pathways, which alleviated the skin changes associated with AD [104]. Luteolin, in preclinical models, inhibits histamine release from mast cells, reducing pruritus and erythema (flush reaction) [31,105]. It reduces TEWL, decreases epidermal thickening, and, in blood studies, a reduction in immunoglobulin E (IgE) and IL-4 was observed [106]. Flavones seem particularly relevant for reducing pruritus and decreasing allergic reactions.

Flavanones include naringenin and hesperidin, which are found in citrus fruits such as oranges, lemons, mandarins, and grapefruits [95,107]. In a murine AD model induced by DNFB, it was shown that naringenin inhibited the JAK2/STAT3 pathway. A decrease in IgE levels in the blood and reduced concentrations of TNF-α, IL-6, IFN-γ, IL-12, and IL-5 in skin lesions were observed [108]. The treatment also reduced epidermal thickness and the number of dendritic cells [109]. Hesperidin, mainly found in citrus fruits, was studied in human keratinocyte cell lines (HaCaT), where it was found to regulate cytokine signaling, which helps protect cells from damage induced by oxidative stress. These data suggest the potential use of hesperidin in the treatment of AD, although further research is needed [110]. Studies by Ikarashi et al. in HaCaT cells suggest that Satsuma mandarin extract may have therapeutic potential in treating dry skin by increasing the expression of aquaporin-3 (AQP3), which improves hydration. Although hesperidin in mandarins was not found to affect AQP3 levels, their anti-inflammatory and antioxidant properties may be valuable in the treatment of AD, where chronic inflammation plays a key role [111]. Flavanones demonstrate immunomodulatory potential primarily through regulation of the JAK/STAT axis.

The most abundant compound in green tea is EGCG (epigallocatechin-3-gallate), which is a representative of flavan-3-ols [112,113,114]. Individuals with AD have an altered skin microbiota composition that is accompanied by increased colonization of *S. aureus*, whose reduction may contribute to decreased skin inflammation [15]. In in vitro studies, EGCG inhibited the proliferation of *S. aureus* and limited biofilm formation. Additionally, EGCG reduced the inflammatory responses induced by membrane vesicles derived from *S. aureus* by modifying the process that incorporates enterotoxin SEA and other proteins into their cargo. This action suggests that EGCG may influence the course of *S. aureus*-dependent inflammation, which has potential significance in diseases such as AD [115]. In AD, there is an increased necroptosis of keratinocytes—a programmed cell death involving protein kinases interacting with receptor 1 (RIPK1), RIPK3, and mixed-lineage kinase domain-like (MLKL) proteins. EGCG in nanoparticle form (mouse model) reduced necroptosis markers and the number of keratinocytes with damaged DNA, suggesting that EGCG may limit keratinocyte necroptosis [116]. Notably, EGCG appears to stand out among polyphenols due to its combined antimicrobial activity against *S. aureus* and its ability to modulate microbiome-related inflammation and keratinocyte necroptosis, which are key processes in AD pathogenesis.

Soy isoflavones, specifically genistein and daidzein, can be converted by specific gut microbiota into equol. In a study by Chiba et al., the level of equol in urine was lower in patients with AD than in healthy individuals, and equol was rarely detected in children with AD. At the same time, no correlation was observed between the level of equol and the severity of skin lesions, suggesting that the potential effect may relate more to susceptibility to AD rather than its severity [117]. In a murine AD model, oral administration of a fermented soy product significantly alleviated skin lesions, reduced epidermal thickening, and reduced eosinophil infiltration and Th2 cytokine expression (IL-5, IL-13), with no effect on IgE antibody levels [118].

Anthocyanins are flavonoids responsible for the coloration of fruits and are found in blueberries, blackberries, black currants, and grapes. The most commonly studied anthocyanins are cyanidin and delphinidin [119]. In an NC/Nga mouse model, which displays AD-like changes, it was shown that an extract preparation containing polyphenols and anthocyanins alleviated skin inflammation, modulated the Th1/Th2 balance, and reduced IL-17 [81]. Available data suggest that the metabolites of soy isoflavones and anthocyanins may influence the immune response in AD; however, clinical studies confirming their therapeutic efficacy are currently lacking.

Overall, different subclasses of flavonoids demonstrate distinct dominant effects: flavonols (e.g., quercetin) primarily support epidermal barrier integrity and inflammatory regulation, flavones are more strongly associated with pruritus reduction, flavanones mainly modulate JAK/STAT-mediated immune responses, while EGCG uniquely combines anti-inflammatory, antimicrobial, and anti-necroptotic properties.

### 5.2. Phenolic Acids and Their Derivatives

Gallic acid, a representative of hydroxybenzoic acids, is present in tea, red wine, vinegar, and some fruits [120]. In mouse models where AD was induced by DNCB, Th17 response activation was observed, while treatment with gallic acid reversed this effect, normalizing the Th17/Treg balance. Studies showed a decrease in the mRNA expression (and protein levels) of cytokines in ear tissue, including IL-4, IL-5, IL-17, and IL-23, as well as a reduction in serum IgE and TNF-α levels [121]. Despite promising experimental data, there are no clinical studies evaluating its efficacy in the treatment of atopic dermatitis.

In addition to hydroxybenzoic acids, hydroxycinnamic acids constitute an important subgroup of phenolic acids. The most commonly studied hydroxycinnamic acids in the context of dermatoses are chlorogenic acid, caffeic acid, and ferulic acid. They are present in coffee, herbs, and red wine [122,123]. Chlorogenic acid (CGA), also known as caffeoylquinic acid, may help alleviate the symptoms of AD [124]. In a cell model using HaCaT keratinocytes, it was shown that CGA reduced the inflammatory response induced by TNF-α/IFN-γ, leading to a significant reduction in the secretion of TARC/CCL17 of about 32% at a concentration of 2 µM and 45% at 4 µM, indicating CGA’s ability to modulate the inflammatory axis characteristic of AD. However, it should be emphasized that these are in vitro data that require further validation in in vivo models and clinical studies [125]. Caffeic acid is present in the leaves of *Orthosiphon aristatus*, known as cat’s whiskers or Java tea. Its immunomodulatory effect is attributed to the presence of caffeic acid in the plant extract, which modulates inflammatory mediators through the modification of nitric oxide synthase (NOS), demonstrating potential in alleviating AD symptoms [126]. Forsythiaside is a phenylethanoid glycoside containing a caffeic acid moiety, classifying it as a derivative of caffeic acid. It exhibits anti-inflammatory effects and antibacterial activity against *S. aureus*, a bacterium that commonly colonizes the skin in AD. Additionally, it inhibits the expression of chemokines, cytokines, and adhesion molecules in keratinocytes, contributing to the alleviation of skin lesions [127]. Ferulic acid is found in wheat bran, tomatoes, and spices. Its ethyl ester has the ability to reduce oxidative stress, which may limit oxidative stress and tissue damage in inflammatory dermatoses [128].

### 5.3. Stilbenes

Resveratrol (RES) is the main representative of stilbenes [129], which naturally occur in grapes, peanuts, mulberries, red wine, and berries and exhibit anti-inflammatory properties [130,131,132]. RES is a compound with antioxidant properties that also reduces UV-induced cell death [133]. It has been shown that RES and pinosylvin enhance the expression of semaphorin 3A (SEMA3A) via the AHR-NRF2 pathway in human keratinocytes. SEMA3A inhibits excessive innervation of the epidermis, which plays an important role in the mechanism of pruritus in AD. Modulating epidermal innervation may represent a new therapeutic target for treating pruritus in AD [134]. RES inhibits the NF-κB pathway and caspase 1, which reduces the expression of TSLP and, in turn, limits mast cell activity. Preclinical studies have shown that topical application of a RES-containing formulation derived from rice reduced pruritus and scratching, decreased the serum levels of IgE and IL-31, and alleviated skin inflammatory changes [135]. Preclinical studies on nasal aerosols in upper respiratory tract infections suggest that combining RES with carboxymethyl-β-glucan increases its bioavailability in an aqueous environment and reduces its degradation, which may contribute to more effective biological activity in topical applications [136]. Pterostilbene is a compound found in grapevines, berries, and peanuts, among others [137]. It has a similar structure to RES, but its advantage is greater bioavailability. In a mouse model of AD, it was shown that topical application of pterostilbene reduced serum IgE levels; decreased the expression of IL-4, IL-6, TNF-α, and inflammatory signaling in the skin; and reduced epidermal thickening in affected mice [138]. Overall, the available data suggest that stilbenes modulate the Th2 response, reduce oxidative stress, and influence epidermal barrier function, making them promising candidates for topical therapy in AD.

### 5.4. Lignans

Secoisolariciresinol is found in flaxseeds, and studies in mice (C57BL/6J strain) suggest that its metabolite, enterolactone (ENL), depending on the concentration, may limit the Th2 response via the JAK-STAT6 pathway. In humans, lower ENL levels negatively correlated with disease severity (SCORAD) [139]. Another lignan with potential therapeutic effects is arctiin, which, in a DNCB-induced AD model, reduced the severity of skin lesions. Its action inhibited the TLR4/MyD88/NF-κB pathway, reduced serum IgE levels, and decreased epidermal thickness and the number of mast cells in the skin. A reduction in the expression of mRNA for TSLP and IFN-γ was also observed. It should be noted that the DNCB model shows only 40% transcriptomic similarity to human AD, so the above results require confirmation in humans [140]. Sesamin, a natural lignan from sesame, exhibits immunomodulatory effects by inducing apoptosis in activated T lymphocytes through MCL-1 dependence. Oral supplementation of sesamin in a mouse model of AD reduced the severity of skin lesions, reduced pruritus, decreased serum IgE levels and Th2/Th17 cytokine expression, and reduced mast cell infiltration, although these results require confirmation in clinical studies. In addition, sesamin induces MCL-1-dependent apoptosis in activated T lymphocytes and alleviates experimental atopic dermatitis [141].

### 5.5. Tannins

Proanthocyanidins (PACs) are condensed tannins that promote wound healing, alleviate inflammatory responses, and exhibit antioxidant effects. PAC modulates the Th2 response, including by increasing the expression of the immunoregulatory cytokine IL-10. The use of PACs reduces eosinophil activity and lowers the levels of IL-4, IL-5, and IL-13 cytokines and IgE. Their anti-inflammatory effect is comparable to that of hydrocortisone, highlighting the potential of PACs as promising compounds in AD therapy [142,143]. The tannin group also includes florotannins, which are polyphenols found in brown algae [144]. These enzymes exhibit immunomodulatory effects by reducing oxidative stress and limiting the action of enzymes such as collagenase and elastase. Limiting their activity reduces collagen and elastin degradation, promoting skin elasticity and improving the healing of skin lesions in AD [145].

### 5.6. Xanthones

Xanthones are found in mangosteen. In a mouse study, it was shown that they reduce pruritus by decreasing the expression of nerve growth factor (NGF) in keratinocytes and fibroblasts. They also exhibit antihistaminic and immunomodulatory effects, including reducing IgE and modulating the Th2 response, which may contribute to alleviating AD symptoms [146]. In an AD model in NC/Tnd mice, oral administration of mangosteen peel extract (250 mg/kg body weight for 6 weeks) led to a decrease in the clinical severity of skin lesions, a reduction in scratching, lower TEWL, and the inhibition of epidermal hyperplasia. In vitro, the addition of the extract to bacterial cultures reduced IgE production and inhibited IgE-dependent mast cell degranulation, while in vivo, a decrease in the mRNA expression of IFN-γ was observed. Moreover, the inhibition of keratinocyte proliferation was demonstrated, supporting the hypothesis of the multifaceted influence of xanthones on the course of AD [147].

Overall, the available evidence suggests that different classes of polyphenols may exert complementary rather than overlapping effects, targeting distinct aspects of AD pathophysiology, including inflammation, epidermal barrier dysfunction, and immune dysregulation.

**Table 2:** Main classes of polyphenols and related bioactive compounds in the context of AD.

Class of Compounds	Representative Compounds	Models (According to Text)	Key Mechanisms	Main Effects in AD/Atopic Dermatitis (AD)	Dominant Effect	Citations
**Flavonols**	Quercetin, Kaempferol, Myricetin	Keratinocytes; BALB/c mice	↓ROS; inhibition of NF-κB; ↓T lymphocyte activation (↓CD69); ↓TSLP; Th2 modulation; restriction of histamine release	↓Inflammatory cytokines (e.g., IL-4/IL-13); ↓TEWL; improvement of epidermal barrier	Barrier support (anti-inflammatory)	[95,98,99,100]
**Flavones**	Apigenin, Luteolin	Keratinocytes; mouse models (BALB/c)	Inhibition of MAPK, NF-κB, and JAK/STAT; ↓histamine release	↓IL-31, ↓IL-33; ↓IgE, ↓IL-4; ↓TEWL; reduction in itching and erythema reaction	Anti-pruritic	[31,101,102,104,105,106]
**Flavanones**	Naringenin, Hesperidin	DNFB mice; HaCaT keratinocytes	Inhibition of JAK2/STAT3; antioxidant action; cytokine regulation	↓IgE; ↓TNF-α, IL-6, IFN-γ, IL-12, IL-5; ↓epidermal thickness; ↓dendritic cells	Immune modulation (JAK/STAT)	[95,108,109,110]
**Flavan-3-ols**	EGCG	Mice; in vitro studies on *S. aureus*	Inhibition of *S. aureus* proliferation and biofilm; restriction of keratinocyte necroptosis	↓*S. aureus*-dependent inflammation; ↓necroptosis markers; ↓DNA damage in keratinocytes	Microbiome modulation (necroptosis regulation)	[115,116]
**Isoflavones**	Genistein, Daidzein → Equol; fermented soy product	Humans (urine); mice	Th2 modulation; JAK-STAT6 (ENL)	Lower equol levels in AD patients; ↓epidermal thickening; ↓IL-5, ↓IL-13; ↓eosinophil infiltration	Microbiome-dependent metabolism	[117,118]
**Anthocyanins**	Cyanidin, Delphinidin	NC/Nga mice	Modulation of Th1/Th2 balance	↓Skin inflammation; ↓IL-17	Th1/Th2 balance (immune modulation)	[81,119]
**Hydroxybenzoic Acids**	Gallic acid	Mice (DNCB)	Normalization of Th17/Treg	↓IL-4, IL-5, IL-17, IL-23; ↓IgE; ↓TNF-α	Cytokine regulation	[120,121]
**Hydroxycinnamic Acids**	Chlorogenic acid, Caffeic acid, Ferulic acid	Mice; HaCaT keratinocytes	Inhibition of Akt1/NF-κB; ↓TARC; NOS modification; ↓ROS	↓Inflammatory response; potential alleviation of AD symptoms	Cytokine/ROS modulation	[122,124,125,126,127,128]
**Stilbenes**	Resveratrol, Pterostilbene	Keratinocytes; mice	Inhibition of NF-κB and caspase-1; activation of the AHR-NRF2 axis (↑SEMA3A)	↓TSLP; ↓IgE; ↓IL-31; ↓itching; ↓epidermal thickening	Neuroimmune/anti-pruritic	[129,134,135,138]
**Lignans**	Secoisolariciresinol (ENL-metabolite), Arctiin, Sesamin	C57BL/6J mice (DNCB); correlation studies in humans	Inhibition of TLR4/MyD88/NF-κB; Th2 modulation; induction of T lymphocyte apoptosis (MCL-1)	↓IgE; ↓TSLP; ↓IFN-γ; ↓itching; ↓mast cell infiltration; ↓skin thickness	T-cell modulation	[139,140,141]
**Tannins**	Proanthocyanidins, Flavotanins	Mice; in vitro studies	Th2 modulation (↑IL-10); inhibition of ROS; ↓collagenase and elastase	↓IL-4, IL-5, IL-13; ↓IgE; ↓eosinophils; improved healing	Th2 suppression	[142,143,144,145]
**Xanthones**	Xanthones (Mangosteen)	NC/Tnd mice; in vitro	↓NGF; antihistamine action; Th2 modulation	↓Itching; ↓IgE; ↓TEWL; ↓epidermal hyperplasia	Anti-pruritic (NGF modulation)	[146,147]

Note: Arrows indicate direction of change (↑ increase, ↓ decrease). Abbreviations: AD—atopic dermatitis; ROS—reactive oxygen species; NF-κB—nuclear factor kappa B; CD69—cluster of differentiation 69; TSLP—thymic stromal lymphopoietin; Th2—T helper type 2; IL—interleukin; TEWL—transepidermal water loss; MAPK—mitogen-activated protein kinase; JAK—Janus kinase; STAT—signal transducer and activator of transcription; IgE—immunoglobulin E; TNF-α—tumor necrosis factor alpha; IFN-γ—interferon gamma; EGCG—epigallocatechin-3-gallate; *S. aureus*—*Staphylococcus aureus*; ENL—enterolactone; Treg—regulatory T cells; TARC—thymus- and activation-regulated chemokine (CCL17); NOS—nitric oxide synthase; SEMA3A—semaphorin 3A; TLR4—Toll-like receptor 4; NGF—nerve growth factor.

## 6. Therapeutic Implications and Outlook

### 6.1. Bioavailability Limitations and Strategies to Enhance Delivery

The bioavailability of polyphenols depends on various factors, including their physicochemical properties and external factors such as soil type, sunlight, substrate moisture, and plant maturity [148]. Moreover, individual factors such as age, health status (especially conditions that impair intestinal absorption), genetic factors, and gut microbiota composition also influence the bioavailability of polyphenols [149]. Polyphenols can interact with the gut microbiota and undergo degradation in the gastrointestinal tract due to the low pH and the presence of digestive enzymes [150]. Most polyphenols are released from conjugated forms in the food matrix during digestion [151]. Ultimately, only 5–10% of polyphenols are transported to the liver [152], with the best absorption seen for isoflavones and gallic acid, and the poorest for anthocyanins [153]. Importantly, the bioavailability of different classes of polyphenols varies significantly, with relatively higher absorption observed for isoflavones and phenolic acids, and markedly lower absorption for anthocyanins and catechins. Additionally, the bioavailability of polyphenols depends on their dietary source and chemical form. For example, onions provide more bioavailable quercetin than apples or tea [154]. Additionally, the optimal delivery system appears to depend on the physicochemical properties of the specific polyphenol, including its polarity and molecular weight. In a previous review of available data, protocatechuic acid showed the highest skin permeation, whereas EGCG showed the lowest; moreover, O/W microemulsions enhanced the delivery of chlorogenic acid, while W/O systems improved intradermal delivery of quercetin [155]. The composition of the meal also seems to play a role. Studies have shown that the interaction between spinach and lemon juice increases the bioavailability of spinach polyphenols, suggesting that lemon juice may support the availability and stability of these bioactive compounds during digestion [156]. In the future, it will also be crucial to determine how food processing methods affect the bioavailability of polyphenols in meals [157,158]. There is a lack of research on non-thermal processing technologies that could effectively support the preservation, extraction, and bioavailability of polyphenols in fruits and vegetables [148,159].

Innovative carrier technologies are currently being developed, such as microgel carriers loaded with naringenin, which could potentially overcome the bioavailability barrier and improve long-term treatment efficacy [160]. Another studied method is encapsulation, which improves the bioavailability of some polyphenols, such as hesperidin, fisetin, and curcumin, while for others, like anthocyanins from blueberries or cocoa phenolic acids, the effects of this method are less consistent [161]. The development of nanotechnology and phytopharmacology may improve the bioavailability and stability of polyphenols, potentially enhancing their therapeutic efficacy in AD while maintaining an acceptable safety profile [162]. Hydrogels may be useful as carriers for topical AD therapy because they allow for controlled delivery of active substances and, due to their high water content, reduce skin dryness. Given the intensity of pruritus, adhesive and self-healing properties are also important, increasing the durability of the dressing on the skin. “Smart” hydrogels are being developed that respond to inflammatory microenvironment conditions (e.g., pH/ROS), which may improve the precision of drug release [163]. However, despite promising preclinical results, the clinical translation of these delivery systems remains limited due to insufficient clinical validation, as well as potential safety and regulatory challenges. These limitations significantly hinder the translation of promising preclinical findings into consistent clinical outcomes, which may partly explain the variability and inconsistency observed in clinical studies on polyphenols in AD. Moreover, the lack of standardized forms of administration (e.g., oral supplements, topical formulations, and advanced delivery systems such as encapsulated or nano-based preparations) and dosing strategies further complicates the interpretation and comparability of clinical results.

In addition to their antioxidant effects, polyphenols modulate mitochondrial homeostasis. Mitophagy, as the selective elimination of damaged mitochondria, is crucial for cellular homeostasis. Its dysfunction can exacerbate oxidative stress and inflammation, promoting dysfunction of the epidermal barrier in AD [164]. Preclinical studies indicate that polyphenols such as RES, quercetin, EGCG, CGA, punicalagin/ellagitannins, and kaempferol can modulate mitophagy, supporting the elimination of dysfunctional mitochondria and reducing oxidative stress [165,166]. In preclinical skin models, quercetin has been reported to promote mitophagy through activation of the SIRT1/PINK1/Parkin pathway, thereby reducing oxidative stress and inflammatory responses [167]. Polyphenols present in green tea, including EGCG, have been reported to enhance mitophagy in dermal fibroblasts and modulate key inflammatory and matrix-remodeling pathways, including TNF-induced MMP-1 expression and ERK signaling; advanced delivery systems may further improve their skin bioavailability [168]. EGCG has been linked to enhanced mitophagy-related signaling, including increased PINK1 and Parkin expression and mitophagosomal formation, in a preclinical mouse model of late-onset Alzheimer’s disease, although these findings were obtained in neural rather than cutaneous tissue [169]. Supporting evidence from a preclinical rat model of hypertension indicates that resveratrol may restore mitophagy through the PINK1-SIRT3 pathway and attenuate endoplasmic reticulum stress and inflammatory signaling in renal and vascular tissues. Although the effects were not examined in the context of AD and were observed to be less pronounced compared with exercise in this model, they support a potential role of polyphenols in regulating mitochondrial quality control [170]. A comprehensive approach combining improved bioavailability, safety evaluation, and identification of new molecular targets, such as the regulation of mitophagy, could allow for more precise and effective use of polyphenols as adjunctive therapy in AD in the future.

### 6.2. Clinical Translation: Are Polyphenols Ready for Adjunct Therapy in AD?

Limited bioavailability is one of the main barriers to leveraging the therapeutic potential of polyphenols, and research is ongoing to address this barrier [160]. Importantly, the biological effects of polyphenols appear to be highly dose-dependent. While low to moderate doses may exert antioxidant and anti-inflammatory effects, higher doses may lead to pro-oxidant activity or adverse systemic effects. Although the doses used are reported in individual studies, particularly in preclinical models, human studies remain highly heterogeneous in terms of compound type, formulation, and administered doses, which limits comparability and prevents the establishment of standardized dosing regimens [116].

Despite these limitations, polyphenol supplementation is increasingly being considered to increase their intake, but the effects of such interventions do not always reflect the benefits observed with a diet rich in natural sources of these compounds. Understanding the interactions between polyphenols and other dietary components is crucial, as health benefits arise not only from the polyphenols themselves but also from their synergy with other substances found in whole food products [171].

The literature still lacks studies evaluating the potential toxicity of specific polyphenols, and further research on their safety is necessary, including dose–response analysis, population differences, and possible interactions with other compounds [172]. Some studies confirm that isoflavone supplementation in men does not cause changes in testosterone or estrogen levels [173]. Some polyphenols, including quercetin, may affect thyroid function by inhibiting the synthesis of thyroid hormones and iodine metabolism [174]. Clinical trial data suggest the possibility of adverse effects during resveratrol supplementation, including gastrointestinal symptoms, headaches, skin reactions, and elevated liver parameters [175]. Polyphenols reduce ROS levels by chelating transition metal ions, such as iron [176,177]. Some polyphenols bind to iron in the intestinal lumen, which may limit its absorption and, in high-risk groups, increase the likelihood of developing iron-deficiency anemia [178]. Polyphenols may also interact with drug-metabolizing enzymes (e.g., cytochrome P450), potentially altering the pharmacokinetics of commonly used medications. Despite generally favorable safety profiles, evidence on population-specific risks remains limited, particularly in pediatric patients, pregnant or lactating individuals, and those receiving immunosuppressive or biologic therapies. These gaps limit the clinical applicability of current findings.

Despite promising preclinical data, clinical evidence regarding polyphenols in AD remains limited, and the lack of standardized doses and supplementation regimens complicates the formulation of definitive recommendations. Moreover, many available clinical studies are limited by small sample sizes, short intervention durations, and heterogeneous outcome measures, which further restricts the generalizability of their findings [179]. Currently, clinical evidence remains limited, and further large-scale randomized controlled trials are needed to evaluate the efficacy of specific polyphenols in patients with AD [31]. Therefore, polyphenols should not be considered as a standalone treatment for AD, although they may serve as an adjunctive therapy for selected patients. Their broader use requires the standardization of preparations, more robust interventional studies, and a more comprehensive evaluation of safety and interactions, especially at higher doses. It should also be noted that, consistent with previous narrative reviews on polyphenols in AD, the available evidence remains highly heterogeneous in terms of study design, interventions, and outcome measures. This heterogeneity, together with the predominance of preclinical data, limits the possibility of performing a quantitative synthesis and highlights the need for more standardized clinical trials. Overall, the clinical efficacy of polyphenols in AD remains inconsistent and limited across available studies. Importantly, this review does not provide a systematic, comprehensive synthesis of all available clinical trials, which further limits the ability to draw firm conclusions regarding clinical efficacy.

## 7. Conclusions

Polyphenols exhibit a multifaceted potential as an adjunctive therapy for AD, as they can affect the integrity of the epidermal barrier, the composition and function of the microbiome, and the chronic inflammatory response. However, the available evidence is largely based on preclinical studies, with relatively limited high-quality clinical data, which affects the strength of the conclusions. The preclinical data and limited clinical evidence suggest that polyphenols may alleviate disease progression by inhibiting the NF-κB and JAK/STAT pathways, modulating the immune response, and reducing oxidative stress (ROS), including neutrophil-dependent processes. Increasing attention is also being given to their impact on mitochondrial homeostasis and mitophagy. Limitations include low bioavailability and variability in responses dependent on the gut microbiota. Preventive, therapeutic, and toxic doses have not yet been defined. The majority of mechanistic insights are based on preclinical models and should be interpreted with caution. Future studies should prioritize establishing safe and effective dose ranges, as well as systematically evaluating potential adverse effects and interactions with commonly used medications. Researchers should focus on several key priorities, including conducting large-scale randomized controlled trials to confirm clinical efficacy, establishing standardized dosing regimens and dose–response relationships, improving bioavailability through advanced delivery systems, clarifying safety profiles and potential interactions, and translating mechanistic findings, such as microbiome modulation and mitophagy regulation, into clinical applications.

## Figures and Tables

**Table 1:** Summary of experimental studies on polyphenol interventions in animal and cell models of AD.

Study (Author, Year)	Mouse Model/AD Inducer	Polyphenol/Intervention	Route of Administration	Main Observed Effects on Skin and Immune System	Proposed Mechanism of Action	Main Limitations
Yang et al., 2023 [79]	hIL-4/hIL-4Rα KI; oxazolone	No specific polyphenol (reference model)	Topical (hapten)	Exacerbated AD features (swelling, inflammation, cellular infiltration)	Model useful for testing biological therapies and Th2-modulating compounds	Hapten model does not capture the multifactorial pathogenesis of AD, dominated by Th2 response, not reflecting the full heterogeneity of AD phenotypes
Ikarashi et al., 2020 [80]	TMA-induced AD	Polyphenols from *Acacia mearnsii*	Oral	↓ Itching, ↓ TNF-α, IL-6, COX-2, iNOS; improvement in skin phenotype	Modulation of the gut–skin axis by changing the gut microbiota	Limited characterization of polyphenol metabolites, lack of direct causal evidence between microbiota change and improvement in AD skin phenotype
Kim et al., 2012 [81]	NC/Nga; DNCB	Polyphenols and anthocyanins from *Vaccinium uliginosum*	Topical	↓ Severity of skin lesions, ↓ inflammation	Anti-inflammatory and antioxidant effects	Chemical model (DNCB) induces strong contact dermatitis, which does not fully reflect atopic dermatitis; short-term observation
Yang et al., 2015 [82]	NC/Nga; house dust mites	Potato epidermis extract (*Solanum tuberosum* cv. Jayoung)	Topical	↑ Filaggrin, improved barrier, better Th1/Th2 balance	Immune response regulation and barrier reconstruction	Lack of standardization of extract and determination of the active compound, no direct assessment of NLRP3/STAT6/NF-κB in skin
Park et al., 2013 [83]	Skin model (in vivo/in vitro experiments)	Resveratrol	Topical/Systemic	↑ Production of cathelicidin	Activation of the S1P (sphingosine-1-phosphate) pathway	Lack of long-term evaluation of epidermal barrier effects, low systemic bioavailability of resveratrol
Saad et al., 2025 (review) [85]	Nrf2−/− mice + *S. aureus*	Polyphenols (various)	Oral	↑ HO-1 and SOD in WT; in Nrf2−/−: ↑ TNF-α, IL-1β, CCL2	Activation of Nrf2 is crucial for the protective action of polyphenols	Heterogeneity of the polyphenols studied
Bangash et al., 2023 [86]	BALB/c; DNCB	Pterostilbene (PTN)	Oral	↓ IgE, ↓ IL-4, IL-6, TNF-α, ↓ NF-κB	Anti-inflammatory and antioxidant effects	Limited analysis of the gut–skin axis, DNCB model does not fully reflect classic AD form
Wu et al., 2020 [87]	BALB/c; DNCB	Phloretin	Oral	↓ Histamine, ↓ IL-6, IL-4, TSLP, IFN-γ, IL-17A	Strong immunomodulation and inhibition of inflammation	Lack of direct assessment of epidermal barrier function, lack of standardization and pharmacokinetic analysis of phloretin
Huang et al., 2025 [88]	BALB/c; DNCB	Oleuropein	Topical	↓ IL-4, IL-5, ↓ COX-2, ↓ IgE, ↓ mast cells and eosinophils	Inhibition of the Th2 axis and allergic inflammation	No assessment of systemic effects
Tang et al., 2025 [89]	DNFB model AD	EGCG	Oral	↓ ROS and MDA, ↑ catalase, ↑ GSH-Px, ↑ HO-1, ↓ TEWL, ↓ IgE	Activation of the Keap1/Nrf2/HO-1 axis	No comparison with reference therapies, no pharmacokinetic data and bioavailability of EGCG

Note: Arrows indicate direction of change (↑ increase, ↓ decrease). Abbreviations: AD—atopic dermatitis; hIL-4—human interleukin-4; hIL-4Rα—human interleukin-4 receptor alpha; KI—knock-in; TMA—trimellitic anhydride; TNF-α—tumor necrosis factor alpha; IL—interleukin; COX-2—cyclooxygenase-2; iNOS—inducible nitric oxide synthase; NC/Nga—NC/Nga mouse strain; DNCB—2,4-dinitrochlorobenzene; Th1/Th2—T helper type 1/type 2; S1P—sphingosine-1-phosphate; Nrf2—nuclear factor erythroid 2-related factor 2; HO-1—heme oxygenase-1; SOD—superoxide dismutase; WT—wild type; CCL2—C-C motif chemokine ligand 2; BALB/c—BALB/c mouse strain; PTN—pterostilbene; IgE—immunoglobulin E; NF-κB—nuclear factor kappa B; TSLP—thymic stromal lymphopoietin; IFN-γ—interferon gamma; DNFB—2,4-dinitrofluorobenzene; EGCG—epigallocatechin-3-gallate; ROS—reactive oxygen species; MDA—malondialdehyde; GSH-Px—glutathione peroxidase; TEWL—transepidermal water loss; Keap1—Kelch-like ECH-associated protein 1.

## Data Availability

The authors used PubMed, ScienceDirect, and Google Scholar databases to screen articles for this review. No new data were generated for this review.

## Abbreviations

The following abbreviations are used in this manuscript:

AD	atopic dermatitis
AMPK	5′ AMP-activated protein kinase
AP	α-hydroxy fatty acid-phytosphingosine
AQP3	aquaporin-3
CGA	chlorogenic acid
BALB/c	Bagg Albino Laboratory strain B/subline c
CCL2	C-C motif chemokine ligand 2
CD69	cluster of differentiation 69
COX-2	cyclooxygenase-2
CXCL1	C-X-C motif chemokine ligand 1
DC	dendritic cell
DNCB	2,4-dinitrochlorobenzene
DNFB	2,4-dinitrofluorobenzene
EASI	Eczema Area and Severity Index
EGCG	epigallocatechin-3-gallate
ELOVL	elongation of very-long-chain fatty acids
ENL	enterolactone
EOS	esterified ω-hydroxy fatty acid-sphingosine
ET	ellagitannins (polyphenolic ellagitannins)
ERK1	extracellular signal-regulated kinase 1
FA	ferulic acid
FFA	free fatty acids
GKS	glucocorticoids
GSH-Px	glutathione peroxidase
hIL-4	human interleukin-4
hIL-4Rα	human interleukin-4 receptor alpha
HO-1	heme oxygenase-1
IFN-γ	interferon-gamma
IgE	immunoglobulin E
iNOS	inducible nitric oxide synthase
IL	Interleukin
JAK/STAT	Janus kinase/signal transducer and activator of transcription
JNK	c-Jun N-terminal kinase
Keap1	Kelch-like ECH-associated protein 1
KI	knock-in
MAPK	mitogen-activated protein kinase
MDA	malondialdehyde
NET	neutrophil extracellular traps
NC/Nga	NC/Nga mouse strain
NF-κB	nuclear factor kappa B
NGF	nerve growth factor
NOS	nitric oxide synthase
NP	α-hydroxy fatty acid-phytosphingosine
Nrf2−/−	Nrf2 knockout/deficient (nuclear factor erythroid 2-related factor 2-deficient)
PAC	proanthocyanidins
PTN	pterostilbene
RANTES	Regulated upon Activation, Normal T-cell Expressed and Secreted (CCL5)
RES	resveratrol
ROS	reactive oxygen species
S1P	sphingosine-1-phosphate
SCFA	short-chain fatty acid
SCORAD	Scoring Atopic Dermatitis
SEMA3A	semaphorin 3A
SOD	superoxide dismutase
SPT	serine palmitoyltransferase
TA	tannic acid
TARC	thymus- and activation-regulated chemokine (CCL17)
TEWL	transepidermal water loss
Th1/Th2	T helper type 1/type 2
Th2	T helper type 2 cells
TNF-α	tumor necrosis factor-alpha
TLR4	Toll-like receptor 4
Treg	regulatory T cells
TMA	trimellitic anhydride
TSLP	thymic stromal lymphopoietin
WT	wild type
